# Premonitory symptoms in migraine: a systematic review and meta-analysis of observational studies reporting prevalence or relative frequency

**DOI:** 10.1186/s10194-022-01510-z

**Published:** 2022-11-12

**Authors:** Anna K. Eigenbrodt, Rune Häckert Christensen, Håkan Ashina, Afrim Iljazi, Casper Emil Christensen, Timothy J. Steiner, Richard B. Lipton, Messoud Ashina

**Affiliations:** 1 Danish Headache Center, Department of Neurology, Rigshospitalet – Glostrup, Faculty of Health and Medical Sciences, University of Copenhagen, Valdemar Hansens Vej 5, 2600 Glostrup, Copenhagen, Denmark; 2 Department of Neurorehabilitation / Traumatic Brain Injury, Rigshospitalet – Glostrup, Copenhagen, Denmark; 3 Department of Anesthesia, Critical Care and Pain Medicine, Beth Israel Deaconess Medical Center, Harvard Medical School, Boston, MA USA; 4 Department of Neuromedicine and Movement Science, Norwegian University of Science and Technology, Trondheim, Norway; 5 Division of Brain Sciences, Imperial College London, London, UK; 6 Department of Neurology and Department of Epidemiology and Population Health, Albert Einstein College of Medicine, Montefiore Headache Center, New York, NY USA

**Keywords:** Prodrome, Epidemiology, Clinical characteristics, International classification of headache disorders

## Abstract

**Background:**

Observational studies on the prevalence of premonitory symptoms in people with migraine, preceding the headache pain (or aura) phase, have shown conflicting results. We conducted a systematic review and meta-analysis to estimate the prevalence, and relative frequency among clinic populations, of premonitory symptoms in people with migraine, overall and of the multifarious individual symptoms, and to review the methodologies used to assess them.

**Methods:**

We searched PubMed and Embase for studies published from database inception until 31^st^ of May 2022. Two investigators independently screened titles, abstracts, and full texts. We retrieved observational studies that reported the prevalence/relative frequency of one or more premonitory symptoms in people with migraine. Two investigators independently extracted data and assessed risk of bias. Results were pooled using random-effects meta-analysis. Our main outcomes were the percentage of people with migraine who experienced at least one premonitory symptom and the percentages who experienced different individual premonitory symptoms. To describe our outcomes, we used the terms *prevalence* for data from population-based samples and *relative frequency* for data from clinic-based samples. We also descriptively and critically assessed the methodologies used to assess these symptoms.

**Results:**

The pooled estimated prevalence in population-based studies of at least one premonitory symptom was 29% (95% CI: 8–63; I^2^ 99%) and the corresponding pooled estimated relative frequency in clinic-based studies was 66% (95% CI: 45–82; I^2^ 99%). The data from clinic-based studies only supported meta-analysis of 11 of 96 individual symptoms, with relative frequency estimates ranging from 11 to 49%. Risk of bias was determined as high in 20 studies, moderate in seven, and low in two.

**Conclusions:**

The substantial between-study heterogeneity demands cautious interpretation of our estimates. Studies showed wide methodological variations, and many lacked rigor. Overall, the evidence was insufficient to support reliable prevalence estimation or characterization of premonitory symptoms. More data are needed, of better quality, to confirm the existence of a distinctive premonitory phase of migraine, and its features. Methodological guidelines based on expert consensus are a prerequisite.

**Supplementary Information:**

The online version contains supplementary material available at 10.1186/s10194-022-01510-z.

## Background

Migraine is a disabling neurological disorder characterized by recurrent attacks of headache of moderate-to-severe intensity and accompanying symptoms such as nausea, photophobia and phonophobia [1, 2]. Some people with migraine also report a symptomatic phase of up to 48 h’ duration “before the onset of pain in migraine without aura and before the aura in migraine with aura” [3]. This phase is referred to as the premonitory (or prodromal) phase of migraine. Interest in this area is motivated by the assumption that investigating the mechanisms of the premonitory phase might clarify the mechanisms of migraine attack initiation [4, 5]. In addition, it has been hypothesized that premonitory symptoms might provide robust attack prediction and thus support the development of pre-emptive therapy [6].

Epidemiological studies have provided estimates of the prevalence of premonitory symptoms in people with migraine [7]. These estimates vary considerably among studies, and it remains unclear whether any specific individual symptoms are characteristic of this phase. This uncertainty hampers experimental investigation of the premonitory phase.

Here, we perform a systematic review of the literature, and meta-analysis, enquiring into the prevalence of premonitory symptoms overall and of individual symptoms. We also describe and critically appraise the methodological quality of included studies, and discuss their limitations. Lastly, we outline directions for future research, with the purpose of improving and standardizing epidemiological enquiry into premonitory symptoms in migraine.

## Methods

### Terminology

The definition of premonitory symptoms (or prodrome) used above is provided by the International Classification of Headache Disorders (ICHD) (Supplementary Table 1) [3, 8–10]. In the four iterations of ICHD so far published, the term *premonitory symptoms* is recommended in the first three (ICHD-1, ICHD-2, ICHD-3β), while the most recent (ICHD-3) recommends the term *prodrome* [3, 8–10]. In an editorial, the incoming Chairman of the Classification Committee, which on behalf of the International Headache Society is responsible for ICHD, expressed a personal opinion that *premonitory symptoms *should be reinstated as the preferred term in future iterations of ICHD [11]. Although this remains an unresolved issue, the two terms are for practical purposes synonymous, describing a symptomatic phase defined as above [3, 8–10]. Since *premonitory symptoms* has been more widely used in the literature, we used this term for the purposes of this review.

To describe the percentage of people with migraine who experience premonitory symptoms, we used the terms *prevalence* when referring to data from population-based samples and *relative frequency* when describing data from clinic-based samples.

### Study selection and data extraction

We followed the Preferred Reporting Items for Systematic Reviews and Meta-Analysis (PRISMA) checklist [12], and registered the study protocol in PROSPERO (CRD42021255339).

We systematically searched PubMed and Embase from database inception until 31^st^ May 2022 for observational studies reporting the prevalence or relative frequency of one or more premonitory symptoms among people with migraine. The search string was “migraine AND (premonitory OR prodromal OR prodrome)”. After removal of duplicates, two of us (AKE and AI), independently, first screened titles and abstracts for relevance, then reviewed the retrieved full texts for eligibility based on pre-defined inclusion and exclusion criteria (Table 1). Eligible studies varied widely in the method of assessing premonitory features and in the extent to which an operational definition was provided. The reference lists of retrieved publications were also searched to identify other eligible studies. Final study selection was determined by consensus between AKE and AI.

**Table 1:** Eligibility criteria for study inclusion

Inclusion criteria	Exclusion criteria
Study participants with a diagnosis of migraine according to the iteration of ICHD in effect at the time	Conference papers, case series, and case reports
Language: English, Danish, German	The terms premonitory or prodrome used as a synonym for or referring to reversible neurological symptoms that can reliably be assessed as aura
Observational studies investigating the overall prevalence or relative frequency of premonitory symptoms in people with migraine (in some cases, also of specific individual symptoms) or observational studies exclusively investigating prevalence or relative frequency of individual premonitory symptoms	Data necessary for the calculation of primary or secondary outcomes cannot be extracted
	Studies reporting results on overlapping cohorts

Two investigators (AKE and RHC) independently extracted data from all studies according to a pre-defined set of variables (Tables 2 and 3), afterwards reaching consensus between them.

**Table 2:** Characteristics and findings of population-based studies included in quantitative analysis reporting prevalence of one or more premonitory symptoms in people with migraine

First author, publication year	Study design	Minimum age of participants (years)	Monthly migraine days	Monthly migraine attacks	Primary endpoint: relative frequency of premonitory symptom(s)	Definition of premonitory symptoms	Enquiry method	Sample size N	Participants with ≥ 1 premonitory symptom n (%)
Baykan, 2015 [13]	Cross-sectional, retrospective	≥ 18	Not reported	6.2/5.5 (with/without allodynia)	No	Not reported	Interview (not specified)	871	587 (67.4%)
Kececi, 2002 [14]	Cross-sectional, retrospective	≥ 7	Not reported	Not reported	No	Not reported	Interview (not specified)	173	82 (47.4%)
Rasmussen, 1992 [15]	Cross-sectional, retrospective	≥ 18	Not reported	Not reported	No	Symptoms occurring days or hours prior to onset of migraine	Patient-completed questionnaire (predefined list of 5 symptoms)	96	13 (13.5%)
Russel, 1996 [16]	Cross-sectional, retrospective	≥ 18	Not reported	Not reported	No	Symptoms occurring days or hours prior to onset of migraine	Patient-completed questionnaire (predefined list of 7 symptoms)	498	39 (7.8%)

**Table 3:** Characteristics and findings of clinic-based studies reporting relative frequency of one or more premonitory symptoms in people with migraine

First author, publication year	Study design	Minimum age of participants (years)	Monthly migraine days	Monthly migraine attacks	Primary endpoint: relative frequency of premonitory symptom(s)	Definition of premonitory symptoms	Enquiry method	Sample size N	Participants with ≥ 1 premonitory symptom n (%)
Gago-Veiga, 2018 [17]	Longitudinal, prospective	≥ 15	Not reported	Not reported	Yes	ICHD-3	Patient-completed diary (predefined list of 29 symptoms)	34	29 (85.3%)
Güven, 2017 [18]	Longitudinal, prospective	Not reported	Not reported	5.0	No	Symptoms before headache phase in ≥ 2 of 3 migraine attacks	Patient-completed questionnaire and headache diary (predefined list of 7 symptoms)	339	143 (42.2%)
Karli, 2005 [19]	Cross-sectional, retrospective	Not reported	Not reported	Not reported	Yes	Not reported	Interview and patient-completed questionnaire (predefined list of 24 symptoms)	56	56 (100.0%)
Kelman, 2006 ([20] p2)	Cross-sectional, retrospective	≥ 16	Not reported	10.0	No	Not reported	Interview (not specified)	1009	360 (35.7%)
Laurell, 2015 [21]	Cross-sectional, retrospective	≥ 5	Not reported	Not reported	Yes	ICHD-3 beta	Patient-completed questionnaire (predefined list of 14 symptoms)	2219	1708 (77.0%)
Quintela, 2006 [22]	Longitudinal, prospective	≥ 14	Not reported	Not reported	Yes	Symptoms day before onset of headache and different from those recorded in questionnaire completed in pain-free period	Patient-completed questionnaire and headache diary (predefined list of 28 symptoms)	100	84 (84.0%)
Santoro, 1990 [23]	Longitudinal, prospective	Not reported	Not reported	Not reported	Yes	Symptoms occurring in at least half of attacks the day before or earlier in same day	Patient-completed diary (predefined list of 20 symptoms)	100	33 (33.0%)
Schoonman, 2006 [24]	Cross-sectional, retrospective	Not reported	Not reported	Not reported	Yes	Symptoms preceding ≥ 2 of 3 attacks (timeframe not otherwise defined)	Patient-completed questionnaire (predefined list of 12 symptoms)	374	335 (89.6%)
Schulte, 2015 [25]	Cross-sectional, retrospective	Not reported	Not reported	10.9	Yes	Symptoms with onset at least 2 h prior to onset of headache	Patient-completed questionnaire and headache diary (predefined list of 27 symptoms)	1010	389 (38.5%)
Schwedt, 2018 [26]	Longitudinal, prospective	≥ 15	Not reported	8.9	No	Not reported	Interview (predefined list of 18 symptoms)	15	15 (100.0%)
Viana, 2015 [27]	Longitudinal, prospective	≥ 18	Not reported	Not reported	No	Symptoms in 24 h before a migraine attack	Patient-completed diary (predefined list of 14 symptoms)	30	13 (43.3%)
Wang, 2021 [28]	Cross-sectional, retrospective	≥ 9	Not reported	Not reported	Yes	ICHD-3	Interview (predefined list of 25 symptoms)	4821	1038 (21.5%)

Any discrepancies during the processes of study selection and data extraction were resolved with the assistance of a third investigator (HA).

### Risk of bias

Two of us (AKE and RHC) independently assessed risk of bias using the Joanna Briggs Institute Critical Appraisal Instrument for Studies Reporting Prevalence Data (Supplementary Table 2) [29]. The instrument contains nine items: (1) Was the sample frame appropriate to address the target population? (2) Were study participants sampled in an appropriate way? (3) Was the sample size adequate? (4) Were the study participants and the setting described in detail? (5) Was the data analysis conducted with sufficient coverage of the identified sample? (6) Were valid methods used for identification of the condition? (7) Was the condition measured in a standard, reliable way for all of the participants? (8) Was there an appropriate statistical analysis? (9) Was the response rate adequate? If not, was the low response rate managed appropriately? Higher total scores indicated lower risk of study bias. Studies were categorized according to the percentage of yes answers as high risk (≤ 49%), moderate risk (50%-69%) or low risk (≥ 70%) [29].

### Statistical analysis

We characterised studies as clinic-based or population-based, and separately analysed data from each. We performed a random-effects meta-analysis, which accounted for between-study heterogeneity and calculated pooled prevalence using the inverse variance method on logit transformed data. Between-study variance was calculated using the restricted maximum likelihood method. The I^2^ statistic was used to assess between-study heterogeneity. I^2^ signifies the amount of variation between studies that can be attributed to study heterogeneity rather than chance. Values ≥ 75% indicate considerable heterogeneity and, therefore, uncertainty surrounding pooled estimates. Meta-analysis was performed only when three or more studies reporting relevant outcomes with a total sample size of *N* ≥ 100 subjects were available. The limited data quantity could not support meaningful meta regressions or funnel plots. All statistical analyses were performed with R version 3.5.2 using the “meta” and “metafor” packages.

## Results

**Fig. 1:**
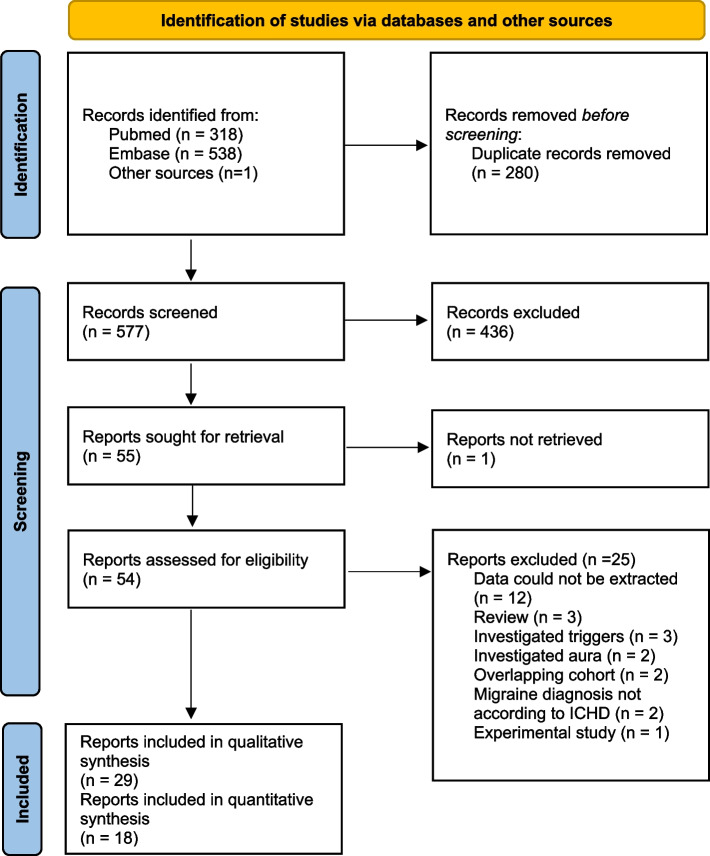
Preferred Reporting Items for Systematic and Meta-analyses (PRISMA) reporting guideline flowchart

**Fig. 2:**
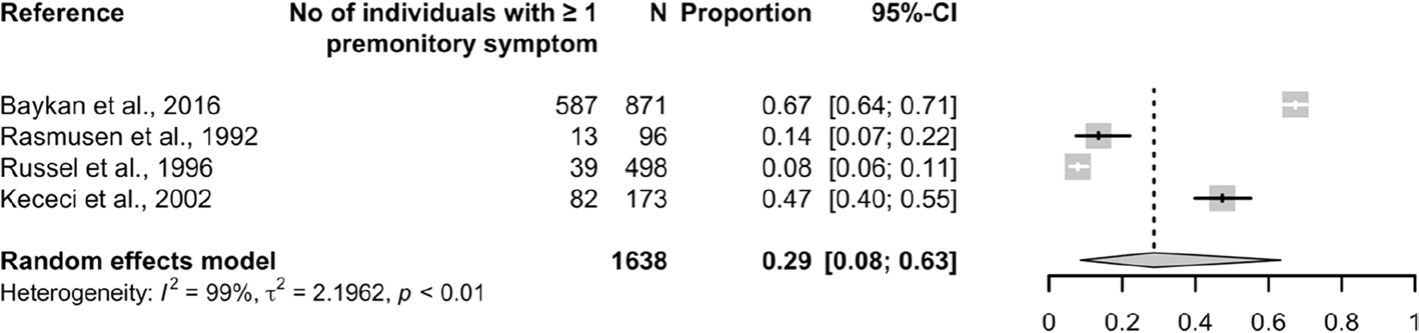
Prevalence of one or more premonitory symptoms in individuals with migraine in population-based studies

**Fig. 3:**
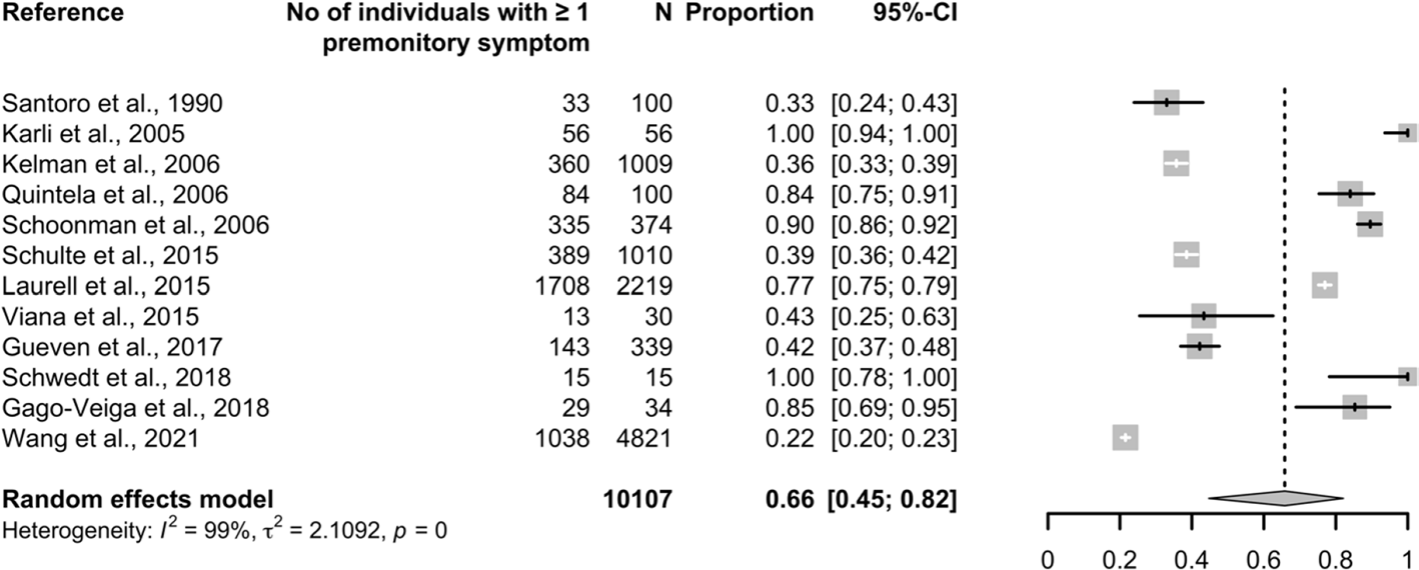
Relative frequency of one or more premonitory symptoms in individuals with migraine in clinic-based studies.

The initial database search identified 857 publications (Fig. 1). After removal of duplicates, we screened 577 articles by title and abstract, selecting 55 for retrieval and full-text review. Of these, 29 (23 clinic-based and six population-based) met the eligibility criteria. Studies included were of two types: those investigating overall prevalence of premonitory symptoms (and, in some cases, of specific individual symptoms), and those exclusively investigating individual symptoms. All 29 studies were included in the qualitative analysis (methodological review), while 18 were included in the quantitative analysis (prevalence and relative frequency estimates). These 18 studies included four population-based studies reporting overall prevalence of premonitory symptoms (Fig. 2, Table 2), 12 clinic-based studies reporting overall relative frequency of premonitory symptoms (in some cases, also of specific premonitory symptoms) (Fig. 3, Tables 3 and 4), and two clinic-based studies exclusively reporting relative frequency of individual premonitory symptoms (Table 4).

**Table 4:** Relative frequency of individual premonitory symptoms in clinic-based studies

Premonitory symptom	Number of studies	Total number of participants	Pooled relative frequency % (95% CI)	I^2^ % (95% CI)
Fatigue	4 [22, 24, 26, 30]	1470	49% (30–68)	80.8% (49.5–92.7)
Neck stiffness	3 [19, 24, 26]	445	46% (20–75)	80.2% (37.7–93.7)
Mood change	3 [26, 30, 31]	1623	37% (10–76)	88.6% (68.7–95.9)
Concentration difficulties	3 [19, 22, 24]	530	30% (25–36)	16.5% (0.0–91.3)
Nausea	4 [22–24, 26]	589	29% (13–52)	85.7% (64.8–94.2)
Photophobia	5 [19, 22, 23, 26, 31]	2153	29% (4–80)	97.6% (96.2–98.5)
Phonophobia	5 [19, 22, 23, 26, 31]	898	26% (4–76)	97.5% (96.0–98.5)
Yawning	7 [18, 19, 22, 24, 26, 30, 31]	2492	22% (7–53)	95.5% (92.9–97.2)
Depressive symptoms	5 [18, 19, 22–24]	969	19% (7–44)	96.2% (93.4–97.8)
Irritability	3 [19, 22, 24]	530	16% (2–65)	86.4% (60.7–95.3)
Food craving	6 [19, 22, 24, 26, 30, 31]	2153	11% (3–32)	89.3% (79.4–94.5)

### Overall prevalence and relative frequency (ie, of at least one premonitory symptom)

Overall prevalence was 29% (95% CI: 8–63; I^2^ = 99%; *N* = 1,638) in population-based studies [13–16] and relative frequency in clinic-based studies was 66% (95% CI: 45–82; I^2^ = 99%; *N*= 10,107) [17–28]. Of the 29 studies included in the qualitative analysis, five exclusively reported pediatric data and were therefore not included in the meta-analysis. Two clinic-based studies estimated overall relative frequency of premonitory symptoms in children with migraine: 42% in one (*N* = 176) [32] and 67% in the other (*N*= 103) [33]. One clinic-based study reported relative frequency in adolescents with migraine (*N*= 19), with follow-up assessments after two years [34]. Premonitory symptoms were reported by three of 19 subjects (17%) at baseline and by nine of 18 (50%) after two years.

### Prevalence and relative frequency of individual premonitory symptoms

Between them, the studies reported a total of 96 specific individual premonitory symptoms. Data were too few from population-based samples, but sufficient to support meta-analysis of 11 of these in clinic-based populations (in order of frequency: fatigue [49%], neck stiffness [46%], mood change [37%], concentration difficulties [30%], nausea [29%], photophobia [29%], phonophobia [26%], yawning [22%], depressive symptoms [19%], irritability [16%], food craving [11%]) (Table 3). Numbers of studies contributing to each analysis were low, and I^2^-values were high (> 85%) for all except concentration difficulties (17%) (Table 3).

### Premonitory versus other phases

Five studies recorded non-headache symptoms during premonitory, headache and postdromal phases [18, 22, 35–37], the last defined in ICHD-3 as occurring in association with a migraine attack but after (up to 48 h) resolution of the headache [3]. All five found that non-headache symptoms commonly reported as premonitory, such as yawning and fatigue, were equally common during the headache and postdromal phases.

### Means of data acquisition and other methodology of included studies

Of the 29 studies, 15 assessed prevalence or relative frequency of premonitory symptoms as the primary outcome [17, 19, 21–25, 28, 31–33, 35, 37–39]. Nine of the 22 clinic-based studies collected data prospectively [17, 18, 22, 23, 26, 27, 35, 36, 39], while 14 [19–21, 24, 25, 28, 30–34, 37, 38, 40], and all six population-based studies [13–16, 41, 42], acquired data retrospectively.

The studies applied different definitions of premonitory symptoms. Six studies used the criteria offered by the ICHD iteration at the time of conduct [17, 21, 28, 33, 37, 39]. Five other studies applied criteria otherwise in accordance with ICHD but specifying time intervals that were shorter or longer than the 48 h interval specified by ICHD-3 [15, 16, 25, 27, 32]. Seven studies used definitions that were not consistent with ICHD [18, 22–24, 35, 38, 40]. Eleven studies provided no definitions [13, 14, 19, 20, 26, 30, 31, 34, 36, 41, 42].

Data were collected by face-to-face interview in 12 studies [13–16, 20, 28, 31–33, 36, 38, 42], by self-administered questionnaires in nine [21, 22, 24, 25, 30, 37, 39–41], by subject-completed diaries in four [17, 23, 27, 35] and by combinations of these methods in four [18, 19, 26, 34]. Eighteen studies used pre-defined lists of 2–29 (from the total of 96) premonitory symptoms [15–19, 21–28, 31–33, 35, 41], four asked specifically about a single specified symptom [36, 37, 39, 40], and seven did not report how they elicited premonitory symptoms. Seven studies using pre-defined lists also included the option of free recall [15–17, 23, 28, 35, 41].

### Risk of bias

We assessed risk of bias as high in 20 studies [14, 17–20, 22, 23, 25–27, 30–36, 38, 39], moderate in seven [15, 16, 21, 24, 28, 37, 42], and low in two [13, 41] (Supplementary Table 3). The majority of studies (24 of 28) used sampling frames that did not appropriately address the target population (Item 1): 23 clinic-based studies focused on migraine patients in the limited context of specialized or non-specialized headache clinics, while one, although population-based, included only specific subgroups of people with migraine. Twenty-three studies recruited participants inappropriately by convenience sampling, judgmental sampling or snowball sampling (Item 2). Sample size appeared inadequate (< *N* = 300) in 15 studies (Item 3). Subjects were not well characterized in 28 studies, with missing data relating to gender, age, proportions with and without aura, monthly headache days and/or monthly migraine days (Item 4). None of the studies used validated instruments to assess premonitory symptoms (Item 6). Five of nine longitudinal studies had high dropout proportions (> 15%); four others did not report dropouts (Item 9). Ten of 20 cross-sectional studies had responder proportions between 60% and 97.4%, while ten did not report responder proportions (Item 9).

## Discussion

To our knowledge, this is the first systematic review and meta-analysis of observational studies reporting premonitory symptoms among people with migraine. We found the pooled relative frequency of these symptoms was 66% in clinic-based studies, while the pooled prevalence in population-based studies was much lower, at 29%. The three most common, symptoms in clinic-based studies were fatigue (49%), neck stiffness (46%) and mood change (37%). Since we observed substantial between-study heterogeneity across all analyses, and most studies showed high (20/29) or moderate (7/29) risk of bias, these estimates should be interpreted with caution.

Several factors might have contributed to heterogeneity among the studies [7, 43]. The nature of the study samples (population vs. clinic-based), the definitions of premonitory symptoms and the methods of ascertaining them differed markedly from study to study. A symptom classified as premonitory in one study might not have been so classified in another. Some studies used retrospective recall while others relied on prospective reporting using diaries. Some studies used clinician interviews while others used self-administered questionnaires. Eighteen studies used pre-defined but varying lists of 2–29 putative symptoms from the cross-study total of 96, some of these studies with the additional option of free recall, four asked only about a single specified symptom and seven did not specify. Clearly, longer lists of putative symptoms increased the probability of reporting one or more, as would the option of free recall. Thus, sources of heterogeneity included sample variance, criteria variance, information variance and interpretation variance [44].

The premonitory phase is defined by being symptomatic. However, it is unresolved whether and with what frequency the same symptoms occur in other phases of migraine. Our meta-analysis found highly non-specific symptoms such as fatigue and mood change to be among the most prevalent of those described as premonitory. These are common symptoms among the general population, and very often bear no association with a migraine attack [45]. The five studies recording non-headache symptoms during premonitory, headache and postdromal phases all found that symptoms commonly reported as premonitory, such as yawning and fatigue, were equally common during the other phases [18, 22, 35–37]. It could be that premonitory symptoms begin before pain but that the process that generates them persists through the headache phase and into the postdrome. If so, these symptoms could still be used to target individuals for biological research or intervention studies. As attack frequency increases the distinctions between premonitory and postdromal symptoms may be blurred. Symptoms between headaches could represent the postdrome of the previous headache or the premonitory phase of an impending headache. According to ICHD-3, a migraine attack accompanied by both premonitory and postdromal phases may last up to seven days [3], so that people with four or more attacks per month may find themselves always in one or other of these phases. Clearly, premonitory symptoms need to be assessed in individuals with a sufficient interval between attacks to resolve prodromes and postdromes. None of the studies reported this variable. Indeed, the majority of studies (23/29) were clinic-based, with participants highly likely to have relatively high-frequency attacks: at least four included people with more than four attacks per month [17, 26, 37, 39], and another five included people with chronic migraine [13, 32, 36, 38, 40].

The risk of conflating what are premonitory symptoms with those of other phases is enhanced by the uncertainties surrounding duration. ICHD-3 defines the premonitory phase as lasting up to 48-h [3], but we did not find empirical evidence to support this. One study reported onset of premonitory symptoms at a mean of 10.6 h prior to the headache phase [17] (p), while another reported 6.3 h [25]. In a third, reporting mean duration as 6.8 h [31], 45% of symptoms lasted less than one hour and only 13% more than 12 h. These data, indicating a generally much shorter-lasting premonitory phase than 48 h, do not support the ICHD-3 definition.

### Strength and limitations

The strength of this study lies in the systematic literature review and meta-analyses following standard (PRISMA) methodological guidelines. The limitations were not in the study itself but in the data. There was considerable between-study heterogeneity largely due to varying and often questionable methodologies. The majority of data came from selected (clinic-based) populations, with any biases this might have introduced (evidence of bias is seen in the different prevalence/relative frequency estimates: 29% population-based and 66% clinic-based). There were sparse data from a limited number of studies (and/or small sample sizes) for some of the meta-analyses and too few data to instigate a meta-analysis on pediatric studies. There were also too few data to permit us to perform meta-regression, which might have clarified the effects on study heterogeneity of different variables such as definition of premonitory symptoms and assessment methods. There were too few data to make a funnel plot to check for publication bias.

### Future directions

Since our findings represent the whole of the available evidence, the key question they generate is whether, in their objectivity or their totality, they confirm the existence of a premonitory phase as a distinct phase of the migraine attack. We are not at all sure they do. This is not to deny that the phase exists: it is an absence of evidence, not evidence of absence. There is work to be done, in five directions.

First, research must address the methodological shortcomings that are evident in the literature, and promote standardisation to make future studies more comparable. Above all, the field needs an operational consensus definition of the premonitory phase. Ultimately, ICHD should be the source of this definition, but our findings suggest that the current definition [3] needs revision and perhaps further specification. Revision requires more and better empirical data than currently exist. Studies designed to acquire these data must carefully consider how best to elicit premonitory symptoms objectively and reliably. Lists of predefined symptoms prompt recall, and are likely to result in higher estimates of prevalence or relative frequency, but they may also lead to false-positive symptom reporting through yea-saying, a well-known phenomenon in survey research [46]. We recommend beginning with open-ended questions, to be followed by lists that include some dummy (highly unfeasible) response options. Ideally, methodological guidelines agreed by expert consensus are needed.

Secondly, studies should be conducted in the population of interest. Clinic-based studies are feasible and of interest to clinicians. Generalizability from speciality care to primary care or from diagnosed patients in primary care to the general migraine population is hazardous. If the goal is to characterize premonitory features in unselected people with migraine, population studies are required. Population-based studies can establish the prevalence of premonitory symptoms and characterize their frequency, intensity and duration. There is, in particular, a need for population-based studies investigating premonitory symptoms in children; all five pediatric studies included in this review were retrospective, clinic-based studies with small sample sizes. To establish premonitory *specificity*, it is also necessary to enquire more closely into the prevalence (and relative frequency) of non-headache symptoms during all migraine phases, including the interictal period. This probably requires prospective studies with contemporaneous diary recordings. Studies that ask participants to recall what might have been premonitory symptoms after they have entered the headache phase, as did the majority of the studies in our review, invite recall error and are likely to introduce bias by reverse causality attribution [7, 47]. Prospective diaries can eliminate these, but only so long as they do not allow *post-hoc *data entry (or amendment) [43]. Unfortunately, this comes at a price: symptoms might be missed because they are not immediately recognised, and because the demands of daily (or more frequent) data entry are onerous, and likely to be neglected. Certain character traits are needed to meet these demands conscientiously, and this in itself introduces bias. The expert consensus guidelines called for above should take a view on this. Prospective diaries support collection of large amounts of data, and examination of multiple factors and any associations between them [7], but there is little benefit if the data are misleadingly unrepresentative.

Thirdly, are premonitory symptoms pathognomic of migraine? To answer this requires capturing pre-headache symptoms in people with migraine and tension-type headache (TTH), and determining which are optimally discriminative between these disorders. One of the included population-based study found that premonitory symptoms were no more frequent in people with migraine compared to those with TTH: the most frequent (low spirits and tiredness) being equally so among both groups [15]. If premonitory symptoms are confirmed as a feature of migraine, enquiry can proceed into how they relate to attack initiation. Are they post-onset, signalling the first beginnings of a migraine attack – a true premonitory phase, or are they pre-onset, epiphenomenal symptoms of a state in which the threshold to attack initiation is lowered?

Fourthly, what are the hallmarks of premonitory symptoms? While some might argue that they should be specific to the pre-headache period, others might argue that the essential hallmark is association, at within-person level, with an increased probability of headache over a defined succeeding period. To implement this definition, prospective diary studies are required, so that within-person risk of headache can be calculated over periods that do and do not follow the recording of a particular symptom or symptom complex. This is the approach that would be most useful to support either biological observation or studies of pre-emptive therapy. Limited work has focused on symptoms that predict future attacks [48].

Finally, population-based studies might enquire into how much premonitory symptoms contribute to migraine-attributed burden. With nothing known of this, it would be an important line of enquiry. Burden-of-migraine studies, including the Global Burden of Disease studies, which rank migraine second among causes of lost health worldwide, have focused entirely on the headache phase of migraine [49, 50].

## Conclusion

This first systematic review and meta-analysis of the prevalence of premonitory symptoms in people with migraine found, in population-based studies, that 29% experience them (or, at least, report them). The proportion is higher (66%) in clinic-based studies, which have in-built bias. These estimates should be interpreted with caution owing to inconsistent definitions of premonitory symptoms, other methodological differences and substantial between-study heterogeneity. Additionally, albeit in only a few studies, symptoms reported as premonitory were equally common during the headache and postdromal phases, with none that were specific to the premonitory phase. Far more data, of better quality, are needed to establish the existence of, and characterize, the premonitory phase of migraine. Methodological guidelines based on expert consensus are a pre-requisite.

## Supplementary Information

**Additional file 1: Supplementary Table 1. **Definitions of terms: “premonitory symptoms” and “prodrome”.** Supplementary Table 2. **Quality assessment of included studies (adapted version of the Joanna Briggs Institute Critical Appraisal Instrument for Studies Reporting Relative frequency Data, updated 2017)**. Supplementary Table 3. **Quality assessment of included studies (adapted version of Joanna Briggs Institute Critical AppraisalInstrument for Studies Reporting Relative frequency Data, updated 2017).

## Data Availability

All data generated or analysed during this study are included in this published article [and its supplementary information files].

## Abbreviations

ICHD: International Classification of Headache Disorders
PRISMA: Preferred Reporting Items for Systematic Reviews and Meta-Analysis
TTH: Tension-type headache
