# Supplementary material for: Premonitory symptoms in migraine: a systematic review and meta-analysis of observational studies reporting prevalence or relative frequency
Source: J Headache Pain. 2022 Nov 12;23(1):140. doi: 10.1186/s10194-022-01510-z (PMC9655795; doi:10.1186/s10194-022-01510-z)
Supplement: Supplementary file 1 — Additional file 1: Supplementary Table 1. Definitions of terms: “premonitory symptoms” and “prodrome”. Supplementary Table 2. Quality assessment of included studies (adapted version of the Joanna Briggs Institute Critical Appraisal Instrument for Studies Reporting Relative frequency Data, updated 2017). Supplementary Table 3. Quality assessment of included studies (adapted version of Joanna Briggs Institute Critical AppraisalInstrument for Studies Reporting Relative frequency Data, updated 2017). [file 10194_2022_1510_MOESM1_ESM.docx]

**Supplementary Table 1.** Definitions of terms: “premonitory symptoms” and “prodrome”.

| Source | Definition of premonitory symptoms | Definition of prodrome |
| --- | --- | --- |
| ICHD-1 (ref) | “Sensations preceding a migraine attack by 2 to 48 hours. Among the common premonitory symptoms are: Fatigue, elation, depression, abnormal hunger, craving for special foods. Occur before the aura or before an attack of migraine without aura.” | “Has been used with different meanings, most often as synonymous with premonitory symptoms. The term should be avoided in the future.” |
| ICHD-2 (ref) and ICHD-3 beta (Ref) | “Symptoms preceding and forewarning of a migraine attack by 2-48 hours, occurring before the aura in migraine with aura and before the onset of pain in migraine without aura. Among the common premonitory symptoms are fatigue, elation, depression, unusual hunger, craving for certain foods.” | “This term has been used with different meanings, most often synonymously with premonitory symptoms. It should be avoided in the future.” |
| ICHD-3 (rEF) | “This term has been used with different meanings, often synonymously with prodrome (qv) but also, less specifically and somewhat ambiguously, for a range of symptoms believed to forewarn of (but possibly the initial phase of) a migraine attack. The term is better avoided.” | “A symptomatic phase, lasting up to 48 hours, occurring before the onset of pain in migraine without aura or before the aura in migraine with aura. Among the common prodromal symptoms are fatigue, elated or depressed mood, unusual hunger and cravings for certain foods.” |
| ICHD-4 alpha (personal opinion) (REF) | “Premonitory symptoms remain the current correct terminology for: The symptomatic phase, lasting up to 48 hours, occurring before the onset of pain in migraine without aura or before the aura in migraine with aura. Among the common premonitory symptoms are fatigue, elated or depressed mood, unusual hunger and cravings for certain foods.” | “… we note that ICHD-3 (4) reversed the definition text of premonitory and prodrome used in previous ICHDs. Users should regard the text as swapped in error, based on the literature.” |

**Supplementary Table 2.** Quality assessment of included studies (adapted version of the Joanna Briggs Institute Critical Appraisal Instrument for Studies Reporting Relative frequency Data, updated 2017)**.**

| Item | Yes | No | |
| --- | --- | --- | --- |
| (1) Was the sample frame appropriate to address target population? | Population-based study with a sample frame representative of the target population. | Clinic-based study or population-based study of specific subgroups of migraine patients (e.g. only including specific migraine phenotype, gender, or age). | |
| (2) Were study participants recruited in an appropriate way? | The study used simple random sampling, stratified random sampling, systematic random sampling or cluster sampling. | The study used convenience sampling, judgmental sampling or snowball sampling. | |
| (3) Was the sample size adequate? | The study had performed a sample size calculation or the study included 300 or more participants. | The study did not perform a sample size calculation or the study did not include 300 or more participants. | |
| (4) Were the study subjects and setting described in detail? | The study reported gender, age, proportions of included subjects with migraine with aura and without aura, monthly headache days, and monthly migraine days. | The study did not report one or more of these variables. | |
| (5) Was data analysis conducted with sufficient coverage of the identified sample? | Inclusion or exclusion criteria provided sufficient coverage of people with migraine. | Inclusion or exclusion criteria resulted in exclusion of subgroups of people with migraine. | |
| (6) Were valid methods used for the identification of the condition? | The study used an ICHD definition of premonitory symptoms and a validated instrument to record premonitory symptoms. | The study did not use an ICHD definition of premonitory symptoms and/or a validated instrument to record premonitory symptoms. | |
| (7) Was the condition measured in a standard, reliable way for all participants? | The condition was measured in the same way for all participants. | The condition was not measured in the same way for all participants. | |
| (8) Was there appropriate statistical analysis? | The study clearly reported numerator and denominator. | The study did not clearly report numerator and denominator. | |
| (9) Was the response rate adequate, and if not, was the low response rate managed appropriately? | Longitudinal studies reported a dropout proportion of <15% or performed a statistical analysis showing no differences between dropouts and non-dropouts. Cross-sectional studies reported a participation proportion of >50%. | Longitudinal studies reported a dropout proportion of >15%, or did not report dropout proportion. Cross-sectional studies reported a participation proportion of <50%, or did not report participation proportion. |  |

**Supplementary Table 3.** Quality assessment of included studies (adapted version of Joanna Briggs Institute Critical Appraisal Instrument for Studies Reporting Relative frequency Data, updated 2017).

|  | Item number in Joanna Briggs Institute Critical Appraisal Instrument | | | | | | | | |  |  |
| --- | --- | --- | --- | --- | --- | --- | --- | --- | --- | --- | --- |
| First author, publication year | **(1)** | **(2)** | **(3)** | **(4)** | **(5)** | **(6)** | **(7)** | **(8)** | **(9)** | **Positive answers** | **Risk of bias: high (≤49%); moderate (50%-69%); low (≥70%)** |
| Baykan, 2015 | Y | Y | Y | N | Y | N | Y | Y | Y | 7 (78%) | Low risk |
| Cuvellier, 2009 | N | N | N | N | Y | N | N | Y | Y | 3 (33%) | High risk |
| Fonseca, 2020 | N | N | N | N | Y | N | N | Y | N | 2 (22%) | High risk |
| Gago-Veiga, 2018 | N | N | N | N | Y | N | Y | Y | N | 3 (33%) | High risk |
| Giffin, 2003 | N | N | N | N | Y | N | Y | Y | N | 3 (33%) | High risk |
| Güven, 2017 | N | N | Y | N | Y | N | Y | Y | N | 4 (44%) | High risk |
| Haytoglu, 2019 | N | N | N | N | N | N | Y | Y | N | 2 (22%) | High risk |
| Ho, 2003 | Y | Y | N | N | Y | N | Y | Y | Y | 6 (67%) | Moderate risk |
| Jacobs, 2019 | N | N | N | N | Y | N | N | Y | N | 2 (22%) | High risk |
| Kallela, 2001 | N | N | Y | N | Y | N | Y | Y | N | 4 (44%) | High risk |
| Karli, 2005 | N | N | N | N | Y | N | Y | Y | N | 3 (33%) | High risk |
| Karsan, 2016 | N | N | N | N | Y | N | N | Y | N | 2 (22%) | High risk |
| Kececi, 2002 | Y | Y | N | N | N | N | Y | Y | N | 4 (44%) | High risk |
| Kelman, 2004 | N | N | Y | N | Y | N | Y | Y | N | 4 (44%) | High risk |
| Kelman, 2006 | N | N | Y | N | Y | N | Y | Y | N | 4 (44%) | High risk |
| Lampl, 2015 | N | N | Y | N | Y | N | Y | Y | N | 4 (44%) | High risk |
| Lampl, 2019 | N | N | Y | N | Y | N | Y | Y | Y | 5 (56%) | Moderate risk |
| Laurell, 2015 | N | N | Y | N | Y | N | Y | Y | Y | 5 (56%) | Moderate risk |
| Pradhan, 2018 | N | N | Y | N | Y | N | Y | Y | N | 4 (44%) | High risk |
| Quintela, 2006 | N | N | N | N | Y | N | Y | Y | N | 3 (33%) | High risk |
| Rasmussen, 1992 | Y | Y | N | N | Y | N | Y | Y | Y | 6 (67%) | Moderate risk |
| Russel, 1996 | N | Y | Y | N | Y | N | N | Y | Y | 5 (56%) | Moderate risk |
| Santoro, 1990 | N | N | N | N | Y | N | Y | Y | N | 3 (33%) | High risk |
| Schoonman, 2006 | N | N | Y | N | Y | N | Y | Y | Y | 5 (56%) | Moderate risk |
| Schulte, 2015 | N | N | Y | N | Y | N | Y | Y | N | 4 (44%) | High risk |
| Schwedt, 2018 | N | N | N | N | Y | N | Y | Y | N | 3 (33%) | High risk |
| Takeshima, 2004 | Y | Y | Y | Y | Y | N | Y | Y | Y | 8 (89%) | Low risk |
| Viana, 2015 | N | N | N | N | Y | N | Y | Y | N | 3 (33%) | High risk |
| Wang, 2021 | N | N | Y | N | Y | N | Y | Y | Y | 5 (56%) | Moderate risk |
